# Supplementary material for: Mothers’ Breast Milk Composition and Their Respective Infant’s Gut Microbiota Differ between Five Distinct Rural and Urban Regions in Vietnam
Source: Nutrients. 2023 Nov 16;15(22):4802. doi: 10.3390/nu15224802 (PMC10675055; doi:10.3390/nu15224802)
Supplement: Supplementary file 1 [file nutrients-15-04802-s001.zip › nutrients-2705276-supplementary.pdf]

Supplementary Tables and Figures

Differences in mother's breast milk composition and their respective infant's gut microbiota between five distinct geo-graphical areas in Vietnam

Table S1: demographics of the cohort sampled from five different regions in Vietnam (mean)

Region	Infant age (days)	Mother age (years)	Mother BMI	Monthly household income (VND; million)	Rural/urban	Typical diet	Typical occupation
Tien Giang	41	29	22.5	5.2	Rural	Fresh water fish	farmer
Phu Tho	38	26	20.4	7.4	Rural	Rice	farmer / worker
Ha Long Bay	40	26	21.9	6.8	Rural	Salt water fish	farmer / worker
Ha Noi	36	28	22.7	10.3	Urban	Western	worker / (small) business
Ho Chi Minh	39	28	23.5	15.9	Urban	Western	worker / (small) business

Table S2: Sample numbers per sample type and region. Columns with sample pairs indicate within family sample dyads.

Region	infant feces	mother feces	mother milk	Infant – breast milk pairs	Infant – mother pairs	Mother – breast milk pairs
Tien Giang	15	15	19	14	15	14
Phu Tho	16	18	22	16	13	18
Ha Long Bay	18	18	16	14	17	15
Ha Noi	13	16	21	13	13	16
Ho Chi Minh	16	15	13	11	15	10

Table S3: Region microbiota characteristics (signature taxa and Faith's phylogenetic diversity)

Region	Typical taxa infant feces	Alpha-diversity (median (IQR))
Tien Giang	*Bifidobacterium, Prevotella, Granulicatella*	10.68 (9.55-11.53)
Phu Tho	*Bifidobacterium, Lactobacillus, Acinetobacter*	9.25 (7.57-11.53)
Ha Long Bay	*Bifidobacterium, Lactobacillus, Propionibacterium*	8.77 (7.95-10.31)
Ha Noi	*Klebsiella, Staphylococcus, Clostridium*	7.86 (7.54-10.08)
Ho Chi Minh	*Blautia, Collinsella, Anaerococcus*	10.18 (8.29-14.18)

Differences in mother's breast milk composition and their respective infant's gut microbiota between five distinct geo-graphical areas in Vietnam

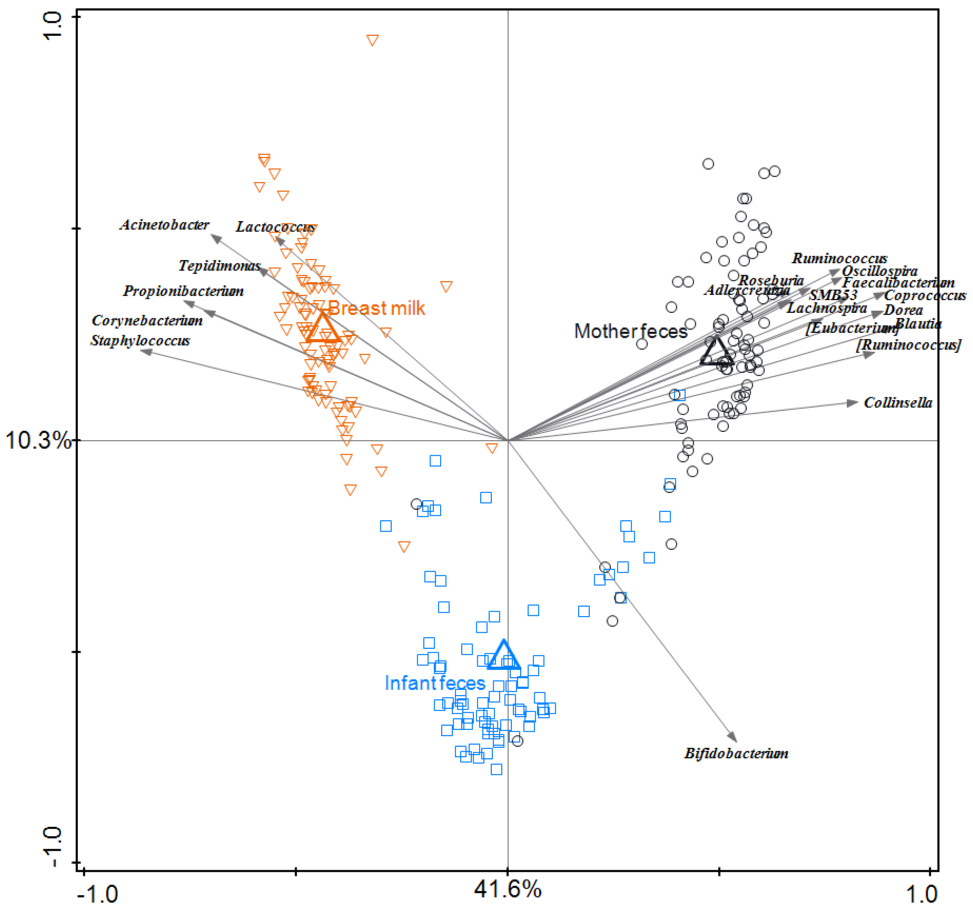

Figure S1: PCA of microbiota relative abundance on the genus level in all samples*. Samples are infant and mother feces, and breast milk from the mothers. Large triangles indicate sample niche centroids and are plotted supplementary. Arrows show the 20 best fitting genera.*

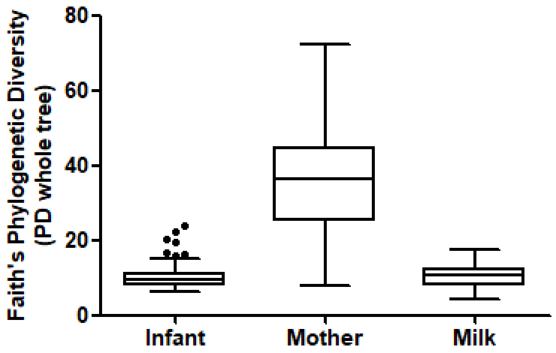

Figure S2: Within-sample diversity differences between sample groups and sample niches. *Columns represent infant and mother feces, and breast milk from the mothers. Boxplots are displayed as Tukey boxplots, Kruskall-Wallis test: p <0.0001*

Differences in mother's breast milk composition and their respective infant's gut microbiota between five distinct geo-graphical areas in Vietnam

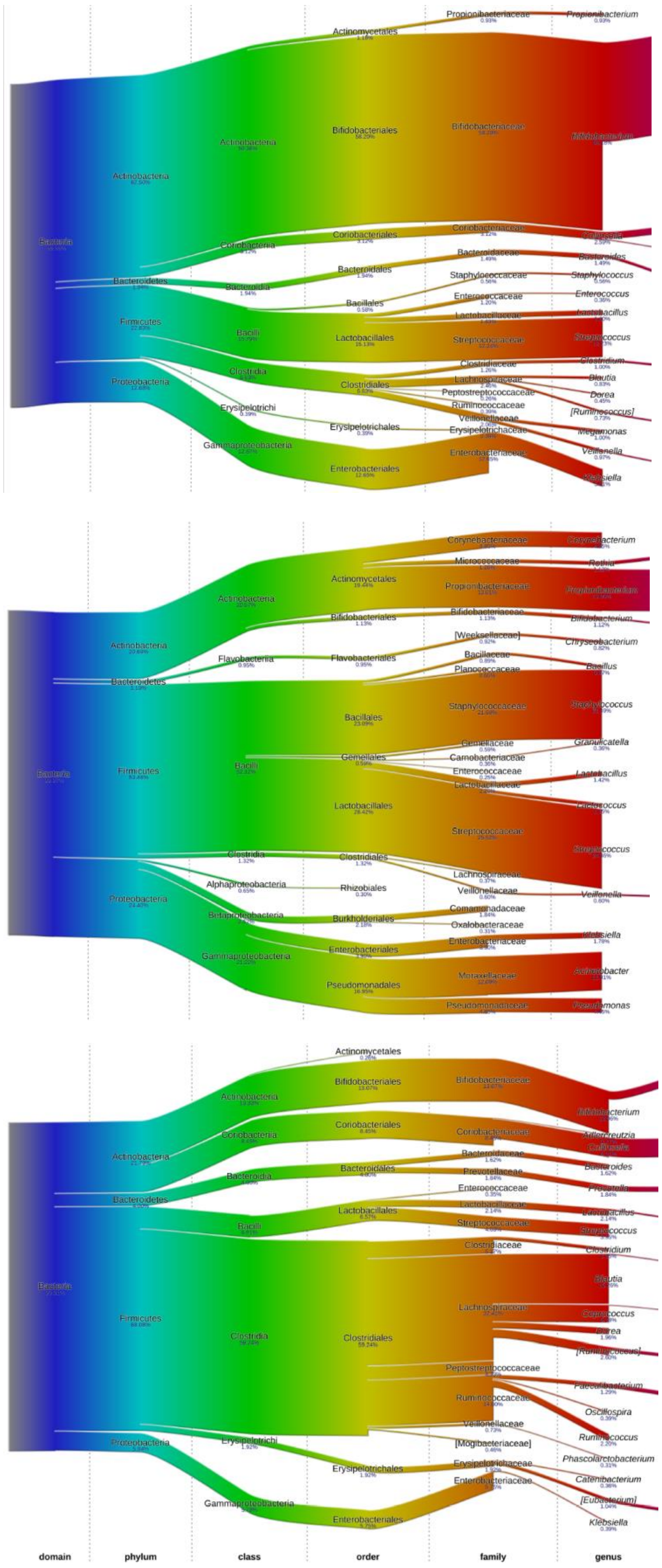

Figure S3: Overall average microbiota composition. Top) *Infant fecal samples.* ***Middle)*** *Breast milk samples.* ***Bottom)*** *Mother fecal samples.*

The fraction of 16S rDNA reads (%) attributed to specific taxonomic levels is given below the taxon name. Figure was generated using software described in Sundquist et al.

Differences in mother's breast milk composition and their respective infant's gut microbiota between five distinct geo-graphical areas in Vietnam

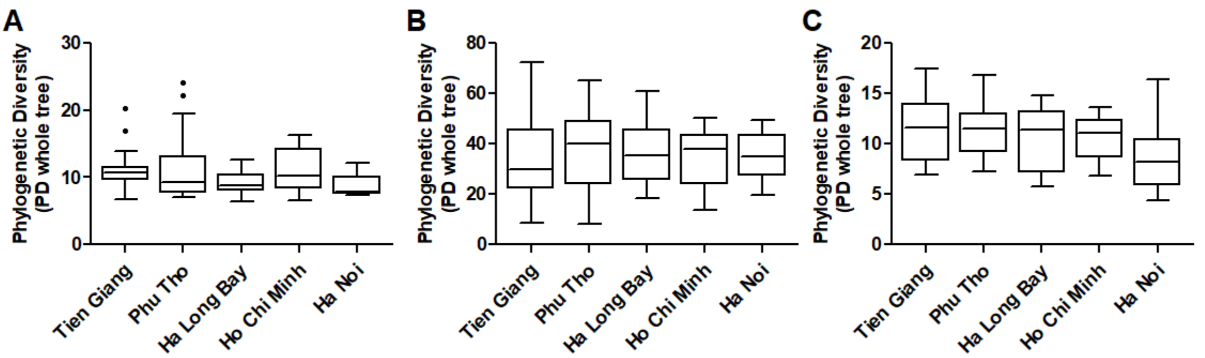

Figure S4: Within-sample diversity (Faith's phylogenetic diversity) differences between regions. A) *Infant feces (p=0.048);* ***B)*** *Mother feces (p=0.922;* ***C)*** *Breast milk (p=0.011). Boxplots are displayed as Tukey boxplots, Kruskal-Wallis test was used to assess overall differences between regions.*

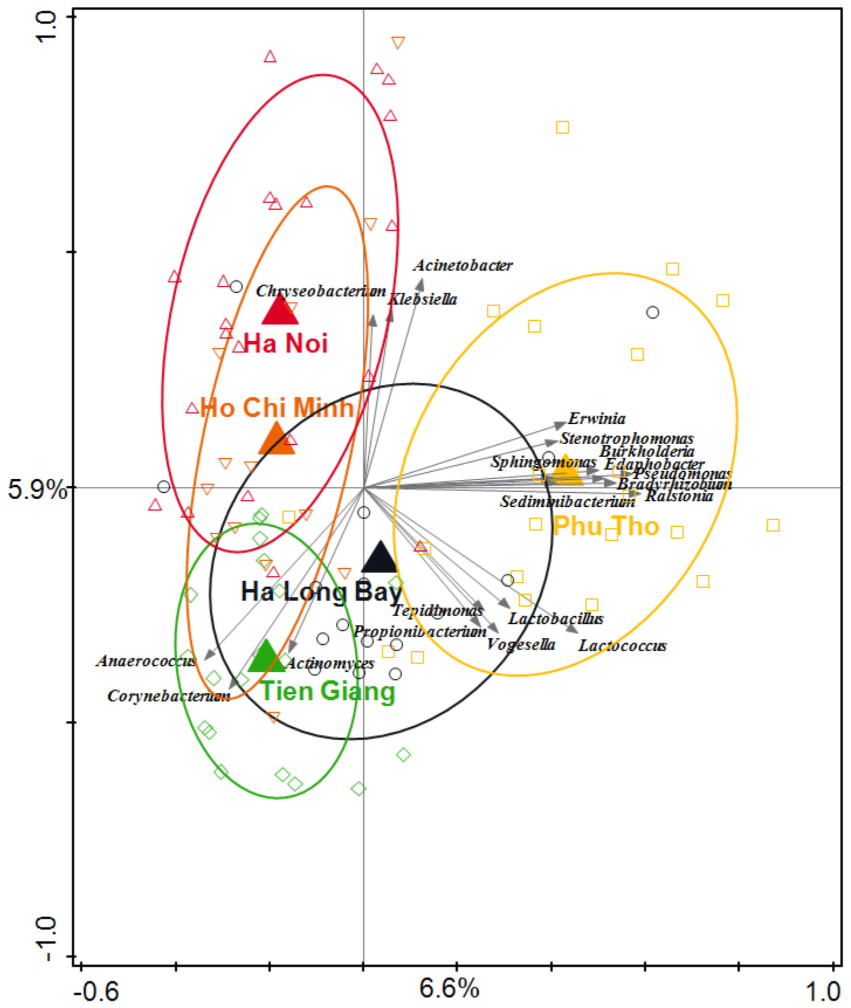

Figure S5 RDA of breast milk microbiota relative abundance on the genus level and geography*.* *Large triangles are centroids of the sample groups (regions), the other symbols indicate individual samples: Ellipses are the 66% quantile of the approximated 2D-Normal density distribution function for each region. Grey arrows are the 20 best-fitting genera. Variation explained by region was 10.4%, p=0.002.*

Supplementary Tables and Figures

Differences in mother's breast milk composition and their respective infant's gut microbiota between five distinct geo-graphical areas in Vietnam

References

Sundquist A, Bigdeli S, Jalili R, Maurice L Druzin Search articles by "Maurice L Druzin" Druzin ML, Waller S, Pullen KM, El-Sayed YY, Taslimi MM, Batzoglou S, Ronaghi M (2007) Bacterial flora-typing with targeted, chip-based Pyrosequencing. BMC Microbiol 7:108. https :// doi.org/10.1186/1471-2180-7-108
